# Discrimination of approved drugs from experimental drugs by learning methods

**DOI:** 10.1186/1471-2105-12-157

**Published:** 2011-05-14

**Authors:** Kailin Tang, Ruixin Zhu, Yixue Li, Zhiwei Cao

**Affiliations:** 1 Shanghai Center for Bioinformation and Technology, 100 Qinzhou Road, Shanghai, 200235, China; 2 College of Life science and Biotechnolog, Tongji University, 1239 Siping Road, Shanghai, 200092, China; 3 Bioinformatics Center, Key Lab of Systems Biology, Shanghai Institutes for Biological Sciences, Chinese Academy of Sciences; Graduate School of the Chinese Academy of Sciences, 320 YueYang Road, Shanghai 200031, China

## Abstract

**Background:**

To assess whether a compound is druglike or not as early as possible is always critical in drug discovery process. There have been many efforts made to create sets of 'rules' or 'filters' which, it is hoped, will help chemists to identify 'drug-like' molecules from 'non-drug' molecules. However, among the chemical space of the druglike molecules, the minority will be approved drugs. Classifying approved drugs from experimental drugs may be more helpful to obtain future approved drugs. Therefore, discrimination of approved drugs from experimental ones has been done in this paper by analyzing the compounds in terms of existing drugs features and machine learning methods.

**Results:**

Four methodologies were compared by their performance to classify approved drugs from experimental ones. The best results were obtained by SVM, in which the accuracy is 0.7911, the sensitivity is 0.5929, and the specificity is 0.8743. Based on the results, consensus model was developed to effectively discriminate drugs, which further pushed the correct classification rate up to 0.8517, sensitivity up to 0.7242, specificity up to 0.9352. The applications on the Traditional Chinese Medicine Ingredients Database (TCM-ID) tested the methods. Therefore this model has been proven to be a potent tool for identifying drug molecules.

**Conclusion:**

The studies would have potential applications in the research of combinatorial library design and virtual high throughput screening for drug discovery.

## Background

In the early 1990s, the advent of high-throughput screening (HTS) and combinational chemistry methodologies was widely seen as having great potential to revolutionize modern drug discovery. However, the quality of the output from these technologies was limited than expected. Despite advances in technology and understanding of biological systems, drug discovery is still a "lengthy, expensive, difficult, and inefficient process" with low rate of new therapeutic discovery [1]. Drugs as well as drug-like compounds are distributed extremely meagerly through chemical space, which is estimated to contain ~10^40 ^to ~10^100 ^molecules. Among the whole chemical space, the majority is nondrug molecules, the minority is druglike molecules. To assess whether a compound is druglike or not as early as possible in drug discovery process will be extremely meritorious. Druglike compounds generally indicates molecules that contain functional groups and/or have physical properties consistent with the majority of known drugs, and hence can be inferred as compounds which might be biologically active or show therapeutic potential [2]. For a drug, properties like synthetic ease, stability, oral availability, good pharmacokinetic properties, lack of toxicity and minimum addictive potential are of utmost importance [3]. Many of these properties depend on the inherent biological and physicochemical parameters of the molecule; whereas the complex structure of the whole drug molecule makes correlating attempts difficult to screen in such a large chemical space. Meanwhile, about more than 80% of all failures of commercial drugs can be attributed to inappropriate absorption, distribution, metabolism, elimination, and toxicity (ADMET) properties despite in vitro and in vivo testing [4-6]. Only a small portion of druglike molecules would survive the rigorous evaluation process and be approved finally, which could be defined as approved drugs. The other compounds are regarded as experimental drugs, which are still in the clinical process or have not been approved for safety and effectiveness yet.

There have been many efforts made to create sets of 'rules' or 'filters' which, it is hoped, will help chemists to identify 'drug-like' molecules from 'non-drug' molecules. The best-known method of drug likeness prediction is the "rule of 5" developed by Lipinski and co-workers [7] by analyzing 2245 available drugs from the World Drug Index (WDI). Ajay et al. [8], Sadowski et al. [9], and Frimurer et al [10] have constructed models to classify druglike and nondruglike molecules, one-dimensional parameters, including molecular weight, ISIS keys (topological indexes) [11], two-dimensional parameters, e.g. functional groups, Ghose and Crippen atom types [12], were used as descriptors. A genetic algorithm-based approach developed by Gillet et al. [13], decision trees used by Wagener et al. [14] have been to distinguish druglike between non-drug compounds. These researches may distinguish compounds that are druglike and nondruglike with good accuracy (about 60%-90%). The most commonly used dataset are listed in Table 1, of which ACD is used as the dataset of non-drugs, and WDI, MDDR, or CMC is used as the dataset for drugs (or drug in development).

**Table 1:** Commonly used datasets

Dataset	Number of compounds
Comprehensive Medicinal Chemistry (CMC) [35]	> 8000 compounds used or studied as medicinal agents
World Drug Index (WDI) [35]	> 80,000 marketed and development drugs worldwide
MACCS-II Drug Data Report (MDDR) [35]	>100,000 drugs launched or under development
Available Chemicals Directory (ACD) [B35]	> 1,160,000 unique chemicals

These above researches have focused on the classification of druglike and nondrug molecules. There are only a little druglike molecules would survive the clinical trials. Discriminating the druglike compound from non-drug molecules is just the first step in long march. Among the chemical space of the druglike molecules, the minority will be approved drugs. Classifying approved drugs from experimental drugs may be more helpful to obtain future approved drugs. However, Discriminations of approved drugs from other molecules have not been reported yet. Can approved drugs be differentiated from experimental drugs? Do the existing 'rules', features and modeling methods still work in the discrimination of approved drugs? In this paper, a further work has been done to assess the molecules which could be marketed drugs rather than experimental drugs. Common used descriptors and classification methods have been utilized to discriminate approved drugs from experimental drugs. In order to evaluate the classification models, the models are applied to a highly possible drug-like database TCM-ID (Traditional Chinese Medicine ingredient Database) [15].

## Methods

### Dataset

Our dataset were downloaded from DrugBank[16] version 2.5. As DrugBank is a resource that combines detailed drug and target information, it contains approved drug and experimental drug. From the original dataset 4554 molecules were processed. The final working set contained 1348 approved drugs and 3206 experimental drugs. The number of compounds per dataset in this study is shown in Table 2.

**Table 2:** The number of compounds per dataset

Dataset	size	Pass Lipinski Rule 5	Pass Oprea rule 3
Approved drugs	1348	1158	1041
Experimental drugs	3206	2621	2271
Herbal ingredients	10370	7599	6058

### Chemical descriptors

Currently various sets of molecular descriptors are available [17]. In order to make approved/experimental classification of compounds, the molecules are typically represented by *n*-dimensional vectors. As the pro-processing, hydrogen was added. The charges and energy optimization of compounds were calculated by Force Field, MMFF94x. The descriptors are calculated by the MOE software (Molecular Operation Environment, version 2008.10). Four sets of descriptors were calculated: 28 druglike index [18] (DLI); 32 widely applicable descriptors [19] (WD); 257 standard MOE descriptors (MOE); 76 Surface Area, Volume and Shape descriptors (SURF). WD descriptors are based upon atomic contributions to van der Waals surface area, log P (octanol/water), molar refractivity and partial charge. The SURF descriptors depend on the structure connectivity and conformation; have been shown to be useful in pharmacokinetic property prediction. All descriptor columns were individually normalized to have a mean of zero and unit variance prior to generation of classification models.

### Classifications Methods

The reported algorithms can be formulated in terms of Machine learning methods. The standard scenario for classifier development consists of two stages: training and testing. During the first stage the learning machine is presented with labeled samples, which are basically *n*-dimensional vectors with a class membership label attached. The learning machine generates a classifier for prediction of the class label of the input coordinates. During the second stage, the generalization ability of the model is tested. Here, four different methods are applied.

### PLSDA

Partial least squares (PLS) is a technique that generalizes and combines features from principal component analysis and multiple regression. Its goal is to predict or analyze a set of dependent variables from a set of independent variables or predictors[20,21]. This prediction is achieved by extracting from the predictors a set of orthogonal factors called latent variables which have the best predictive power. PLS regression is one of the most powerful data mining tools for large data sets with many variables with high collinearity.

### KPLS

KPLS was first described by S. Wold [22] and applied to spectral analysis in the late nineties. Rosipal[23] introduced KPLS in 2001 as a nonlinear extension to the linear PLS method. This nonlinear extension of PLS makes KPLS a powerful machine learning tool for classification as well as regression. KPLS can also be formulated as a paradigm closely related (and almost identical) [24] to Support Vector Machines (SVM).

### ANN

An artificial neural network (ANN), often called as "neural network" (NN), is a mathematical model or computational model based on biological neural networks. It consists of an interconnected group of artificial neurons and processes information using a connectionist approach to computation [25]. In most cases an ANN is an adaptive system that changes its structure based on external or internal information that flows through the network during the learning phase.

### SVM

Support Vector Machines work by mapping the training data into a feature space by the aid of a so-called kernel function and then separating the data using a large margin hyperplane. Intuitively, the kernel computes a similarity between two given examples. Most commonly used kernel functions are RBF kernels. More details on SVMs have been provided in the literature numerous times [26,27].

### Model evaluation and validation

To assess the ability of these four classification methods to predict new chemicals, five-fold cross-validation was used. 20% of chemicals were randomly chosen as the test set; the remaining 80% were used to generate the models. The test set was not used in any way to influence the training and selection of the models. For each five-fold validation, the random experiment was repeated 10 times independently. Accuracy (Ac), sensitivity (Sn), specificity (Sp) and coefficient correlation (CC) are often used to evaluate prediction systems. Sn, Sp and Ac are expressed in terms of true positive (TP), false negative (FN), true negative (TN), false positive (FP) rates:

## Results and discussions

### Classification results

**Figure 1:**
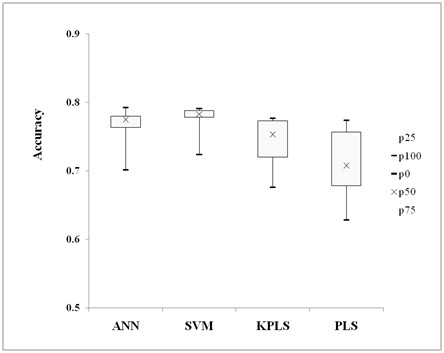
**Results of classification**. Boxplot of performance for the four classification methods.

In order to compare the classification ability of the four classifiers, the results of different descriptors on different models have been scanned. The results distribution of the four classification methods were displayed graphically in Figure 1. The median accuracy were 76.54%, 77.86%, 72.61%, and 69.14% by ANN, SVM, KPLS and PLS respectively. From Figure 1, it can be seen that the results of ANN and SVM were better than those of the KPLS and PLSDA. SVM gives stable performance; the next robust one is ANN. The accuracy distributions of KPLS and PLSDA are sparser and the results are not as robust as ANN and SVM. The classification performance of SVM was slightly better than ANN, significantly outperformed KPLS (p = 0.0224) and PLS (p = 0.0090). SVM has also been compared to ANN and linear discrimination analysis for drug and non-drug datasets previously [28,29]. Results presented here are generally in agreement with these previous observations.

Due to robust convergence behavior SVM seems to be well-suited for solving binary classification problems especially with large variables. In previous studies, SVM performed better than ANN when large numbers of features or descriptors are used [30]. But it is not observed in this paper.

**Figure 2:**
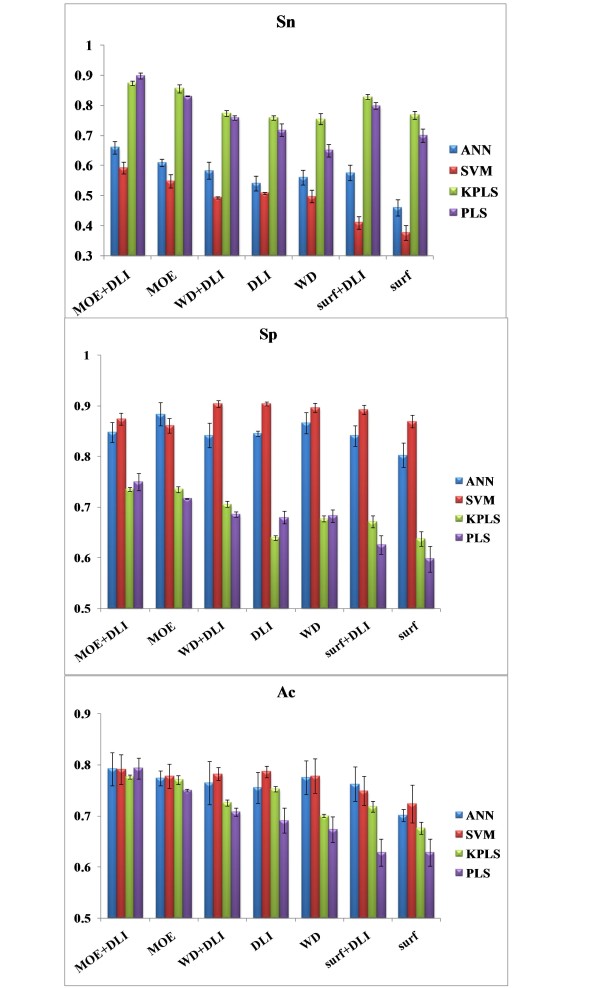
**Results of cross-validation**. Histograms to illustrate the Sn, Sp, and Ac of 10 times 5-fold cross-validation

Since the WD and SURF descriptors are subsets of the standard MOE descriptors, seven sets of descriptor combinations are used for classification. The classification results of four methods and seven sets of descriptors are shown in Figure 2. As reported before [28], 77.89% and 80.19% correction rates were obtained by ANN and SVM respectively in classifying drugs and nondrugs using the standard MOE descriptors. In this paper, using MOE descriptors with ANN and SVM classifiers gave classification rates of 77.47% and 77.85% in discrimination of approved and experimental drugs. The rank order of descriptor sets with regard to the overall classification accuracy yielded was as follows: MOE+DLI, MOE, WD+DLI, DLI, WD, surf+DLI, surf.

MOE descriptors contained 2D and 3D descriptors. 2D molecular descriptors are defined to be numerical properties that can be calculated from the connection table representation of a molecule (e.g., elements, formal charges and bonds, but not atomic coordinates). There are two types of 3D molecular descriptors: those that depend on internal coordinates only and those that depend on absolute orientation. The descriptor number of MOE is far more than that of other methods. From the result of our study, the more comprehensive the descriptors is, the better results are obtained. While the descriptors were chosen on the basis of simplicity, ease of calculation, and diverse representation of chemical properties, simple descriptors are popular in research. Among these descriptors used in this study, the DLI maybe made fairly important contribution that additional descriptors were unlikely to significantly improve prediction accuracy. Considering the complexity of hundreds of thousands of descriptors, such generic and simple chemical properties are so predictive. These simple descriptors have been shown previously to encode and have been used successfully in the past to predict diverse datasets [31,32]. WD descriptors are applicable descriptors and the results of it are in the median of the best and worst. The SURF descriptors led to approximately 10% lower accuracy than the best one. The SURF descriptors have been shown to be useful in pharmacokinetic property prediction but not take effect in this case.

The fundamental problem of the method is how to characterize samples with precise and informative features. From the above results, MOE descriptors conformed well in approved and experimental drugs classification. The DLI descriptors, which made fairly important contribution, were employed to characterize molecules due to its calculation based upon the knowledge derived from known drugs.

### Consensus modeling

In this study, it is noticed that the classified results of different descriptors on different models various largely. For example, the number of approved drugs and experimental drugs which were correctly classified by over 60% methods was 38.5%, 79.1% on average respectively. The molecules are correctly classified in some models while misclassified in others, which indicates their complementary to each other.

Thus we proposed jointly applications of all predictive systems. One possibility to combine several estimators is to employ a voting, e.g. calculating an ensemble average. The other is to construct a consensus model. The central idea of the consensus model is to use the results of multiple, heterogeneous classifiers with variables which may maximum the diversity of the classifiers as the input variables in a new layer classification.

Each classification method has its own strengths and weakness; the ensemble of similar classifiers would inherit such benefits and drawbacks. The four classification methods used in this paper have different advantages, it is useful to construct a consensus model by summarizing different pattern [33]. Here, the obtained classifiers' results in above section are fed into the second layer SVM to get the final result. The results are listed in Table 3.

**Table 3:** classification results

	Sn	Sp	Ac	CC
Best SVM	0.5929	0.8743	0.7911	0.5077
Voting model	0.5523	0.9320	0.8197	0.5415
Consensus model	0.7242	0.9352	0.8517	0.6449

From the results in Table 3, the consensus model gained widely improvement and outperformed the other methods, such as the best SVM and the voting model. The order of accuracy yielded was as follows: consensus model, best SVM, voting model. Compared to the results of best SVM, the sensitivity of consensus model increased 13%, the specificity increased 6% and the accuracy increased 6%. Compared to the results of voting model, the sensitivity of consensus model increased 17% and the accuracy increased 4%. The sensitivity means true positive, that is to say, correctly classified approved drugs. For example, an approved drug--Sulfinpyrazone is misclassified by best SVM and voting model as experimental drugs, while it is discriminated correctly using consensus model. The specificity means true negative. Here it means classifying the experimental drugs correctly. An experimental drug--Adenosine-5-Diphosphoribose, which is misclassified as approved drugs by SVM and voting model, is correctly classified by consensus model. The vote scheme is usually tend to accept the prediction with more voting supports, which may ignore the special samples. This limits the prediction accuracy. While in the consensus model, the results of first level classifiers are used as the input of the second layer SVM, which will avoid unnecessary voting and can combine the results of different methods. The consensus model would further improve the prediction accuracy and robustness of a predictor.

### Application to herbal ingredients

Herbal ingredients have been expected as a potential druglike database. The utility of natural products as sources of novel structures is still alive and well. In the area of cancer, over the time frame from around the 1940s to date, of the 155 small molecules, with 47% actually being either natural products or directly derived therefrom[34]. A comparison by Feher and Schmidt [35] showed that, overall, natural products are more similar to drugs than compounds obtained from combinatorial synthesis. A large proportion of natural products is biologically active and has favorable ADME/T properties (absorption, distribution, metabolism, excretion, and toxicology).

Since the major properties were similar, we used the model constructed by approved drugs and experimental drugs to test herbal ingredients. The final model was applied to TCM-ID. The results showed that 3726 compounds were classified as potential drugs from 10370 molecules. While about 58% and 73% ingredients passed Lipinski 5 rules filter and Oprea filter respectively.

In order to verify the discrimination results, there are three kinds of compounds listed in Table 4. Type I is the intersection of herbal ingredients and approved drugs, type II is the intersection of herbal ingredients and experimental drugs and type III is unknown compounds. 76% compounds in typeI all passed the filter by our model, while 80% passed Lipinski 5 rules and 66% compounds passed the Oprea 3 rules. About 22% compounds in typeII were misclassified as drugs by our model while 79% compounds were misclassified by Lipinski 5 rules and 66% were misclassified by filter of the Oprea 3 rules. From the above results, our model is comparable to Lipinski 5 rules and Oprea 3 rules when they are use to predict a compound as a candidate drug. Our model is better than the others when they are used to justify a compound as nondrug. The model would be useful to narrow down the space of drug prediction and screening.

**Table 4:** Predicted results

Compound type	Compound number	Pass Lipinski 5 rules	Pass Oprea 3 rules	Pass Our model
typeI	59	47	39	45
typeII	68	54	45	15
typeIII	10243	7498	5974	3666

Compounds in typeIII are unknown to us for whether they would be a candidate drug. The passed compounds by different filter rules are different. For example, Aristolochic acid has been proved being carcinogenicity and high nephrotoxic and may be a causative agent in Balkan nephropathy. It passed the filter of Lipinski 5 rules and Oprea 3 rules while it was taken as an experimental drug in our model. Astragaloside IV, which is a main ingredient in many Chinese patent medicines, is predicted as a candidate drug in our model while not pass the filter of Lipinski 5 rules and Oprea 3 rules. Whether it is a potential drug or not will be tested by further experiments.

## Conclusions

From the work, discrimination of approved drugs and experimental drugs is practicable. A comparison of four widely used classification methods has shown that, on average, the SVM is able to generate the most predictive models to discriminate approved and experimental drugs, followed by ANN, KPLS and then PLSDA. Seven sets of molecular descriptors were applied to the discrimination of approved drugs and experimental drugs. Notably, these descriptors have comprehensible definitions and physicochemical meanings for drug properties. The classifiers have been complement to each other. The correct classification rate is up to 85.17% by using the consensus model. The herbal ingredients dataset was tested, indicating that this database is a good source for drug discovery. Furthermore, It will not only narrow down the space of drug prediction and screening but also facilitate drug discovery, which the approved drugs and experimental drugs' discrimination has been implemented into the early stage of drug discovery by discarding compounds that are likely to fail further down the baseline.

## Authors' contributions

KLTang performed the classification, prediction and wrote the manuscript, RXZhu performed the molecules pre-processing and calculated the descriptors by MOE. YXLi participated in the study design. ZWCao supervised the study. All authors read and approved the final manuscript.
